# Why time flies? The role of immersion in short video usage behavior

**DOI:** 10.3389/fpsyg.2023.1127210

**Published:** 2023-04-14

**Authors:** Yurong Yan, Yingfei He, Longfei Li

**Affiliations:** ^1^School of Journalism and Communication, Northwest University of Political Science and Law, Xi'an, China; ^2^School of Media and Communication, Shanghai Jiao Tong University, Shanghai, China

**Keywords:** short video, immersive experience, technological affordance, usage behavior, novelty effect, entertainment value, irritation value

## Abstract

**Background:**

Short videos are becoming increasingly popular globally, and users are devoting more time to viewing them. However, few studies have examined the characteristics of short video content and the technical features that are related to media use. The present study developed a model to explore the influence of technological affordances on short video usage and considered innovation in terms of format, setting, and content.

**Method:**

A total of 496 viewers of short videos from China were surveyed. The participants completed 31 Likert-scale items. The study used maximum likelihood estimation modeling.

**Results:**

The results revealed that perceived novelty and content value (entertainment and irritation) affected immersion positively, consequently influencing intentions for reuse and recommendation. The ease of navigating an application, narrative structure, and information value had no significant effect on immersion.

**Conclusion:**

These findings have confirmed that perceived situation affordance and the affective affordance of short videos in digital environments that are managed by intelligent algorithms. It is necessary to analyze the potential impact of different affordances specifically.

## 1. Introduction

Short video development has been significantly facilitated by the development of 5G communication technology, efficient network transmission rates, and the increasing popularity of certain forms of hardware. According to Cisco, short videos may have accounted for 82% of all internet traffic in 2022 (Cisco, 2018). The short video has attracted a large number of users and is becoming increasingly popular. Notably, the Chinese market has grown explosively in recent years. The number of short video users in China reached 975 million in December 2021, and the average daily usage exceeds 2 h (CNNIC, 2021). TikTok, which is owned by the Chinese firm Byte Dance, has more than 600 million users, making it one of the most influential and widely used social media platforms in the world (Iqbal, 2021). Its popularity was the main reason for our decision to conduct this study within a Chinese context.

However, the popularity of short videos has also given rise to numerous concerns related to browsing behaviors, regarding time consumption, ego depletion, and even pathological internet use (Elhai et al., 2017; Yang et al., 2021). Most scholars have focused on internet access and the effect of varying levels of media usage (Kelly et al., 2018; Paez et al., 2020; Hwang and Nam, 2021). However, they have ignored the differences among various applications (Ling and Lai, 2016), especially the technological affordances that affect positive feelings and behaviors (Huang et al., 2022). It has been shown that structural features of media messages (e.g., camera changes, animations, voice changes, sound effects) and personally salient stimuli (e.g., information relevant to an ongoing goal) elicit orienting responses in media users (Lang, 1990; Lee and Lang, 2015). Therefore, we examined the influence of technological affordances on the use of short videos. Short videos have the following obvious characteristics: They run for less than 10 min and mainly consist of segments that last between 60 s and 3 min (von der Osten, 2021; Mileva, 2022). These videos tend to present compact narratives, enabling users to grasp important ideas. In addition, short videos are displayed in vertical formats that differ from the widescreen formats of movies, television, and traditional video platforms. The vertical format reflects the habits of users and the visual design of smartphones (Ryan, 2018). Their low technical requirements allow most users to upload original content and browse videos that they find interesting. In this way, they can acquire practical skills and follow live shows (Jason, 2019).

The media often refer to addictiveness when they cover short-video consumption (CYOL, 2021). However, this proposition focuses entirely on the length of use. Conversely, the concept of immersion denotes the involvement of an individual in a task environment rather than in the natural world. The individual becomes unaware of the passage of time (Jennett et al., 2008). Immersive experiences have been defined as highly enjoyable psychological states (Sarkar and Khare, 2018). Researchers have examined such experiences on various media platforms (Ahn et al., 2016; van Krieken, 2018), including in the context of short videos (Wang, 2020). In short video streaming, an artificial intelligence (AI) algorithm recommends fresh content based on personal browsing habits and interests. Short video streaming is thus different from other social networking applications (Weimann and Masri, 2020; Xu and Tayyab, 2021). Short video platforms provide endless access to content through innovations. Although immersive experiences have been explored in certain studies, the focus has mainly been on the technical environment of a given medium, and few attempts have been made to investigate the influence of the content features of short videos (Cummings and Bailenson, 2016).

Why does time fly? Short video browsing behavior reflects preferences for the information flows that intelligent digital algorithms create. Little is known regarding the characteristics of short video content and the technical features that are related to media usage. Our study has explored the social effect of technological affordances through quantitative research methods. It thus differs from previous qualitative analyses of the concept and is intended to improve the contemporary understanding of the impact of social media on the behavior of users of short videos in the era of digital media.

## 2. Literature review

### 2.1. Immersive experiences and usage behavior

The concept of immersion originated in a study of virtual reality technology. Foley (1987) was the first to mention the problem of immersion in virtual reality, suggesting that immersion would represent the future of the technology. An immersive environment calls for the creation of a panoramic simulation that improves user experience and heightens the effect of media (Foley, 1987). The notion of an immersive experience is well-known in virtual gaming. For example, Brown and Cairns (2004) found that immersed gamers invest time, effort, and attention in games and became less aware of themselves and their surroundings; they even feel isolated from reality. In a broader sense, immersion indicates a lack of awareness of time and the natural world. The concept denotes involvement and a sense of existing within a task environment (Jennett et al., 2008). Immersion has been shown to be related to factors associated with games, for instance, the music used in a game and the screen size of devices on which a game is played (Cairns et al., 2014). Immersion is possible on various platforms, including print (Green et al., 2004), television (Wijnants et al., 2017), virtual reality (Ahn et al., 2016), and multimedia (van Krieken, 2018).

The effect of an immersive experience on behavior has been confirmed in several studies. An immersive experience enhances preferences for specific spaces, services, and/or experiences, such as theme parks (Waysdorf and Reijnders, 2016), museums (Damala et al., 2013), and movies (Fornerino et al., 2008). Use intention and frequency of media usage have been shown to result from immersion in a mobile e-service (Ltifi, 2018) and on the Facebook platform (Rodríguez-Ardura and Meseguer-Artola, 2019). One study has suggested that the use of a bullet screen, which is called “DanMu” in China, affects immersion on video websites and enhances intentions to revisit and to provide positive word of mouth (Fang et al., 2018). As mobile short-form videos were being developed on social media applications, some scholars began to study immersion in that context (Wang, 2020).

The present study has explored two types of user behavior, namely the tendency to continue using short videos (Lin and Lu, 2011) and the tendency to influence other users (Fang et al., 2018), that is, to provide positive word of mouth. Previous studies have also referred to these behavioral intentions to assess loyalty (Hwang and Kandampully, 2012), which is essential for product or service innovation. Furthermore, Shao and Lee (2020) have explained that TikTok relies on satisfaction and intention to induce continuous usage. This study focused on user behaviors that immersion triggers when short content is presented continuously, especially in the context of videos on mobile applications. Consequently, the first hypothesis was formulated as follows:

*H1*: Immersive experiences in short videos are positively related to (a) intention for continued use and (b) positive word of mouth.

### 2.2. Immersive experience hypothesis

Slater and Wilbur (1997) have suggested that immersion is a characteristic of technology. They have emphasized that a system that removes participants from the real world is more likely to lead to immersion. Such systems typically offer inclusive, extensive, all-encompassing, and vivid illusory environments as well as accurate body mapping and self-contained narratives. Affordance theory posits that a characteristic of an object or the environment affords organisms opportunities to perform actions (Gibson, 1986). Moreover, affordance is a perceived property of a thing that determines how the thing could be used by a human (Norman, 1990). The concept of technological affordance captures the manner in which objects (including digital technologies) provide goal-oriented actors with opportunities for action (Markus and Silver, 2008). The attributes and abilities of users, the materiality of technologies, and the contexts of technology use can be dynamic, and the concept provides a framework for probing the relationships among them (Evans et al., 2017). Increasing digitalization has highlighted the value of discussions regarding technological affordances (Lu et al., 2014; Shin, 2017), which can be defined as the features of a digital technology that may trigger certain behaviors that users recognize. The term encompasses content, form, and modes of human-computer interaction that are common in digital media and have been shown to enable immersion, enjoyment, and temporal dissociation experiences (Huang et al., 2022).

Agrawal et al. (2020) identified three causes of immersion: a subjective sense of being absorbed or of experiencing multisensory stimulation, absorption in a narrative or a depiction of a narrative, and absorption in strategic or tactical challenges. Primary studies that have drawn on meta-analyses have indicated immersion-related features that are mostly technical in nature, such as tracking level, the field of view, stereoscopic vision, and sound quality (Cummings and Bailenson, 2016). Emotional content has been identified as one of the few content features that are relevant (Cummings and Bailenson, 2016), which is why we explore it in this study. The hypotheses that we have advanced consider the affordances that are related to media content and the technological properties of short videos.

#### 2.2.1. Ease of navigation

Navigability is considered important for understanding user experience in the context of certain interfaces. Navigability is related to the physical affordance of an information technology artifact that users can sense and act upon (Zhao et al., 2013). For example, the speed and convenience with which users can find content and information is an integral aspect of web design (Sundar and Limperos, 2013; Bedi et al., 2017), in that rapid reactions lead to tactical immersion (Agrawal et al., 2020). Research has found that perceived ease of navigation induces emotional and behavioral responses. A website that is seen as easy to navigate is also perceived as being more pleasant to use (George and Kumar, 2013). Such a website induces a stronger sense of flow (Hsu et al., 2013), and its design encourages impulse purchases (Lin and Lo, 2016). Regarding dynamic images, Guo et al. (2021) found that navigation could affect immersion and enjoyment among digital museum visitors. Meng and Leung (2021) further found that navigability causes short videos to become more satisfying to users. Navigability is a vital part of the online experience of users (including users of short video platforms), and it is connected to search functionalities. It thus triggers continuance intentions (Gong et al., 2018) and promotes electronic word of mouth (Dutta and Bhat, 2017). In line with the literature presented above, we formulated a second hypothesis:

*H2*: The perceived ease of navigating a short video is positively related to immersive experience.

#### 2.2.2. Novelty effect

Absolute and relative novelty have been examined in past research. The former term refers to objects that have never been seen before. The latter describes a familiar object in a new place or an encounter with a novel arrangement of familiar objects (Mendelson, 2001). The novelty effect is more significant for incidental encoding than for intentional encoding (Kormi-Nouri et al., 2005). From the perspective of cognitive psychology, novel items are recognized more easily than familiar ones (Tulving and Kroll, 1995). The synthetic offerings of digital media are considered to be fluid and indeterminate processes of meaning creation, which are different from the enabling and constraining outcomes of inherently positive affordances (Siegert et al., 2020). In the current research, the characteristics of short video applications, such as vertical presentation and unique interfaces, can be seen as being novel and preventing attention from being diverted away from the application (Agrawal et al., 2020). Moreover, because the content is presented at random, users generally cannot anticipate subsequent videos, which may arouse their curiosity. Thon (2008) has described those who focus their attention on unfolding stories as experiencing temporal immersion.

Gaming research has focused on media platforms. One common finding has been that the presentation of content that is perceived as novel and involves unexpected, unusual, and rare events leads to immersion (Liu and Shiue, 2014). Novel digital platforms also lead to more intense emotional responses, as measured by skin conductance (Turner-McGrievy et al., 2013), as well as a greater willingness to access content (Brown, 2002). Consequently, we formulated a third hypothesis:

*H3*: The perceived novelty of a short video is positively related to immersion.

#### 2.2.3. Narrative structure

Narrative affordances are related to the opportunity for a future story to be presented to an audience in the form of media content (Cardona-Rivera and Young, 2013). Structural affect theory postulates that three elements (suspense, surprise, and curiosity) result in emotional responses and account for the degree to which one enjoys narration (Brewer and Lichtenstein, 1982). A satisfactory narrative structure is considered to give full play to gamers’ subjectivity, to control their emotions, and to strengthen their senses of substitution, immersion, and accomplishment (Li and Kim, 2020). Empirical research has confirmed the influence of narrative affordances on subjects’ affective and cognitive processing. The related literature mainly has examined the structural effects of stories in print media. For example, it has been found that a suspenseful narrative structure arouses more emotion and results in more immersion (de Graaf and Hustinx, 2011) and in a feeling of being “taken away” from the story world (Gerrig, 1993). Likewise, a story that contains a surprise is appreciated more and remembered more accurately. Curiosity also affects readers’ enjoyment (Hoeken and Vliet, 2000). Finally, it has been found that repeated exposure to suspenseful films can result in affective habituation or desensitization to repeated stimuli (Chun et al., 2020).

It is worth noting that new media and computer-mediated communication have resulted in a re-examination of narrative theory. Textual features are related to the length, style, and genre of content (Bazzanella, 2010). Researchers have indicated that regarding perceived ease of navigation, the effectiveness of a 360-degree video advertisement presenting a narrative differs from that of a standard narrative video advertisement in terms of transportation, promotional effectiveness, and emotional responses (Feng, 2018). A short video might attract more attention if its structure is suspenseful, surprising, or probing, which can enhance its effect during continuous playback. In light of the above observations, we formulated a fourth hypothesis as follows:

*H4*: The narrative structure of a short video is positively related to immersion.

#### 2.2.4. Content value of short videos

As mentioned previously, the primary antecedents of immersion are related to its technical aspects. Little attention has been paid to features such as emotional content (Cummings and Bailenson, 2016). Perceived cognitive affordance and perceived affective affordance are considered to be two key components of the interactive design of social media. The former is related to users’ perceptions of social media as supporting conceptual, analytical, and problem-solving processes, while the latter refers to the attributes of artifacts that can trigger emotional reactions (Zhao et al., 2013).

Previous research has suggested that perceived advertising value influences the behavior of audiences. Ducoffe (1995) examined three factors, namely informativeness, entertainment, and irritation, to evaluate advertising value, which in turn can influence consumer responses (Ducoffe, 1995). As web advertising evolved, Ducoffe developed measurement scales and confirmed the applicability of the three factors. The term “informativeness” refers to the capacity of an advertisement to provide information about alternative products. “Entertainment” refers to its potential to fascinate consumers. “Irritation” is related to annoyance and offense that consumers may experience (Ducoffe, 1996). The roles of these factors have also been examined in the context of advertising materials on mobile social network services (Ha et al., 2014), on YouTube (Dehghani et al., 2016), and on similar platforms. Most of these studies have reported significant results. Moreover, those results have indicated that there is a positive relationship between informativeness, entertainment, and the efficacy of advertisements. Irritation is negatively related to efficacy (Ducoffe, 1995, 1996). These factors may attract or divert users’ attention, and they are likely to affect immersion (Thon, 2008; Agrawal et al., 2020).

We applied the analytical logic and the findings from the literature outlined above regarding advertising value to short videos. Our analysis accounted for informativeness, entertainment, and irritation. We examined the perceived content value of short videos. A survey of short video users revealed that interesting videos, that is, videos that induce positive feelings and those that describe skills, attract considerable attention (Zhen, 2018). In the literature, direct and indirect relationships between advertising value and purchasing intention have emerged as meaningful. Therefore, our study considered whether immersion could be affected by perceived content value. In line with previous work, we developed the following research question (RQ): Do (a) information value, (b) entertainment value, and (c) irritation value exert influences on the immersive experiences of users?

We used a stimulus–organism–response framework, which originated from environmental psychology. External environmental stimuli affect the cognitive or emotional states of individuals (organisms) and result in behavioral responses (Mehrabian and Russell, 1974). This model has been applied extensively to computer-mediated communication in order to understand user behavior and online media consumption (Luqman et al., 2017; Fu et al., 2018). Technically related factors that affect both social media and online commerce platforms (perceived ease of use, perceived usefulness, visibility, interactivity) have been considered as stimulus cues in previous studies (Hwang and Cho, 2018; Tuncer, 2021). Our study involved four stimuli, namely (1) perceived ease of navigation (physical affordance), (2) the novelty effect (situation affordance), (3) narrative structure (narrative affordance), and (4) the content value of short videos (cognitive and affective affordance). We attempted to understand the relationships between immersive experiences and certain technological affordances of short videos as well as to explore the manner in which an immersive experience can shape intentions for return and recommendation.

## 3. Method

### 3.1. Sample

We collected data from WenJuanXing (http://www.wjx.cn), a Chinese commercial online survey service provider that is similar to MTurk, between January 1 and March 31, 2019. The WenJuanXing platform allowed us to distribute the questionnaire through WeChat, the most popular mobile instant messaging service in China, which was particularly appropriate for recruiting younger individuals (Mei and Brown, 2018). The data collection protocol was approved by the institutional review board of the university with which the authors are affiliated. An introduction explaining the purpose of the research was placed at the beginning of the questionnaire. All participants provided written informed consent and were informed of their rights of withdrawal, confidentiality, and anonymity prior to taking the survey. Qualified participants were identified by means of a filtering question; those who had never used short video applications were disqualified.

A total of 526 individuals completed the questionnaire, and 496 of them submitted valid responses. We conducted a seriousness check by excluding participants with exceedingly short completion times (Aust et al., 2013). A total of 30 invalid questionnaires were discarded because of the low time spent (answering the questions in less than 90 s) (Gu et al., 2022). Among the participants, 62.3% were female and 37.7% were male, 40.1% were aged between 19 and 25 years, and 32.5% were aged between 26 and 35 years. The average age of the participants was 30.31 years (*SD* = 12.03). Regarding education levels, 48.6% of participants held or were completing a bachelor’s degree, and 36.3% were pursuing a master’s degree or another postgraduate qualification.

In terms of exposure to short video applications, 63.9% of the respondents reported watching short videos on applications at least several times a week, and 33.3% used such services several times per day or more frequently. Most of the participants were acting as passive recipients of content (92.7%), which was in accordance with the focus of this study. Some participants were heavily involved in short video applications; for example, some would share their own videos (9.7%) or interact with others (23.0%) on the platforms.

### 3.2. Process and measurements

This study involved 31 items that were developed in line with previous studies and revised to assess the extent to which participants agree with items after watching short videos. For example, the sentence “The features of Foodies.com seem unique to me” (Kim and Gambino, 2016) was revised to “The features of short video apps seem unique to me”; the sentences that began with “Advertisements are…” (Ducoffe, 1995) were revised to “Short videos are…” All scale items were closed-ended, and participants were asked to assess their preferences on a seven-point Likert scale (1 = “strongly disagree,” 7 = “strongly agree”). The items were translated from English to Chinese with the aid of two bilingual Ph.D. students. Back-translations were used. After comparing the different versions, we modified the Chinese translation to ensure that its constructs were accurate. Two professors who were native speakers of English and Chinese confirmed the meanings of the items. Next, 30 graduate students pretested the questionnaire. Table 1 displays the complete set of measurement items. In addition to the Likert scales, the survey also accounted for demographic variables (e.g., age, gender, and individual income per month) and habits (e.g., frequency of use, preferred type of content, and preferred mode of viewing).

**Table 1:** Measurement development.

Construct	Item code	Measurement items	References
Immersive experience	IM1	Watching most of the content on short video apps creates an immersed sense.	Cuny et al. (2015)
	IM2	Watching most of the content on short video apps causes me to lose awareness of my surroundings.	
	IM3	Watching most of the content on short video apps makes me forget the reality of the outside world.	
	IM4	Watching most of the content on short video apps makes me forget my immediate surroundings.	
	IM5	Watching most of the content on short video apps creates a new environment that suddenly disappears at the end of the video.	
Ease of navigation	EON1	Most short video apps are user friendly.	Feng (2018)
	EON2	There are visual cues and/or auditory cues that guide me through the viewing experience.	
	EON3	During navigation, I know where the desire area of focus is.	
Perceived novelty	PN1	Most of the content on short video apps is original.	Ang et al. (2007) and Kim and Gambino (2016)
	PN2	Most of the content on short video apps differs from my expectations.	
	PN3	Most of the content on short video apps is memorable.	
	PN4	Most of the content on short video apps is novel.	
	PN5	The features of short video apps seem innovative to me.	
	PN6	The features of short video apps seem unique to me.	
Narrative structure	NS1	I find most of the content on short video apps suspenseful.	Hoeken and Vliet (2000)
	NS2	I find most of the content on short video apps surprising.	
	NS3	Most of the content on short video apps makes me curious.	
Information value	InV1	Short videos are good sources of information for me (e.g., daily tips and news).	Ducoffe (1995)
	InV2	Short videos provide relevant information (e.g., daily tips and news).	
	InV3	Short videos provide timely information (e.g., daily tips and news).	
Entertainment value	EV1	Short videos are enjoyable.	
	EV2	Short videos are fun to use.	
	EV3	Short videos are exciting.	
Irritation value	IrV1	Short videos insult people’s intelligence.	
	IrV2	Short videos are annoying.	
	IrV3	Short videos are irritating.	
Positive word of mouth	WOM1	I intend to encourage friends to visit the short video apps that I like.	Moon et al. (2013)
	WOM2	I plan to say positive things about the short video apps that I like to other people.	
Reuse intention	RI1	I intend to use short video apps in the future.	Zhou et al. (2012)
	RI2	Given the opportunity, I will use short video apps in the future.	
	RI3	It is likely that I will continue using the short video app that I like in the next few months.	

The measurement model was evaluated by reference to a maximum likelihood estimate (MLE), which was suitable for the task at hand (Myung, 2003). All hypothesized relationships and exploratory questions were tested by using Mplus (version 7.0; Muthén and Muthén, 1998/2013). To include relevant control variables, we followed previous studies that have shown that age influences immersion and intentions to return and to recommend (Cuny et al., 2015).

## 4. Results

### 4.1. Measurement model

We performed confirmatory factor analysis (CFA). In this way, we pre-determined the structural model and tested the hypothesis test. Specifically, we chose five fit indices to estimate the model fit, namely model chi-squared, the comparative fit index (CFI), the Tucker-Lewis index (TLI), the root mean square error of approximation (RMSEA), and standard root mean square residuals (SRMR). The CFA results showed appropriate fit (as demonstrated in Table 2), and every fit index reached the recommended values (Bentler, 1992; Hu and Bentler, 1999).

**Table 2:** Goodness-of-fit indices of the measurement model.

Fit index	χ^2^/df	CFI	TLI	RMSEA	SRMR
Recommended value	<3	>0.9	>0.9	<0.08	<0.08
Measurement model	2.69	0.94	0.93	0.06	0.05

To test the fit of the measurement model, we checked the reliability of each construct by using Cronbach’s alpha test and composite reliability (CR) values. Each value ranged between 0.78 and 0.93, exceeding the 0.7 thresholds suggested by Hair (2010). Therefore, the measurement scales were reliable. We investigated convergent validity by reference to average variance extracted (AVE) and factor loading. The value of the former was at the recommended level and higher than 0.5 (Fornell and Larcker, 1981). In addition, the factor loadings of most items approached 0.7, and all values were larger than 0.5 (Hair, 2010). Therefore, convergent validity was confirmed (see Table 3).

**Table 3:** Convergent validity and reliability statistics.

Construct	Items	Factor loading	S.E.	*t*	*P*	AVE	CR	Cronbach’s *α*
Immersive experience	IM1	0.71	0.03	27.58	0.00	0.66	0.91	0.91
	IM2	0.87	0.02	56.30	0.00			
	IM3	0.83	0.02	46.93	0.00			
	IM4	0.84	0.02	48.79	0.00			
	IM5	0.80	0.02	40.36	0.00			
Ease of navigation	EON1	0.63	0.03	19.30	0.00	0.55	0.79	0.78
	EON2	0.79	0.03	31.22	0.00			
	EON3	0.79	0.03	31.44	0.00			
Perceived novelty	PN1	0.67	0.03	25.01	0.00	0.59	0.90	0.90
	PN2	0.75	0.02	32.87	0.00			
	PN3	0.76	0.02	34.14	0.00			
	PN4	0.83	0.02	48.99	0.00			
	PN5	0.80	0.02	42.24	0.00			
	PN6	0.80	0.02	41.00	0.00			
Narrative structure	NS1	0.74	0.03	28.67	0.00	0.58	0.80	0.79
	NS2	0.84	0.02	38.24	0.00			
	NS3	0.69	0.03	23.40	0.00			
Information value	InV1	0.78	0.02	34.11	0.00	0.63	0.84	0.83
	InV2	0.83	0.02	40.92	0.00			
	InV3	0.77	0.02	32.25	0.00			
Entertainment value	EV1	0.83	0.02	40.44	0.00	0.68	0.87	0.90
	EV2	0.80	0.02	35.95	0.00			
	EV3	0.84	0.02	42.47	0.00			
Irritation value	IrV1	0.76	0.02	34.79	0.00	0.73	0.89	0.89
	IrV2	0.95	0.01	73.30	0.00			
	IrV3	0.85	0.02	51.16	0.00			
Positive WOM	WOM1	0.78	0.03	30.12	0.00	0.66	0.80	0.80
	WOM2	0.84	0.02	34.43	0.00			
Reuse intention	RI1	0.93	0.01	95.03	0.00	0.81	0.93	0.93
	RI2	0.87	0.01	67.33	0.00			
	RI3	0.90	0.01	79.60	0.00			

CR, composite reliabilities; AVE, average variance extracted.

Furthermore, we investigated discriminant validity in order to check for differences between the latent constructs. All items should load highest on their respective constructs. Thus, the square roots of the average variances shared by the items of a construct should be more significant than the coefficients of correlation among the different constructs within the model. We obtained a satisfactory result (Fornell and Larcker, 1981) that demonstrated appropriate discriminant validity (see Table 4).

**Table 4:** Correlations among the latent constructs.

	IM	EON	PN	NS	InV	EV	IrV	WOM	RI
IM	0**.81**															
EON	0.37	0**.74**													
PN	0.61	0.49	0**.77**											
NS	0.55	0.37	0.71	0**.76**									
InV	0.40	0.59	0.62	0.46	0**.79**							
EV	0.51	0.65	0.67	0.50	0.65	0**.83**					
IrV	0.16	−0.10	−0.03	0.27	−0.10	−0.21	0**.86**			
WOM	0.41	0.34	0.60	0.45	0.56	0.52	−0.08	0**.81**	
RI	0.28	0.40	0.52	0.33	0.54	0.60	−0.21	0.68	0**.90**

Diagonal elements (the bold values) show the square root of the AVE of each construct.

We tested for common method variance (CMV) by following Harman’s one-factor method. All measurement items were used for factor analysis, and seven factors were extracted under principal component analysis when eigenvalues were not rotated (Livingstone et al., 1997). The total variance explained was 71.71. The variance of the first factor was 36.07, lower than 40% (Hair, 2010). Therefore, CMV was not problematic, and we continued to test the structural equation model.

### 4.2. The structural model testing

**Figure 1:**
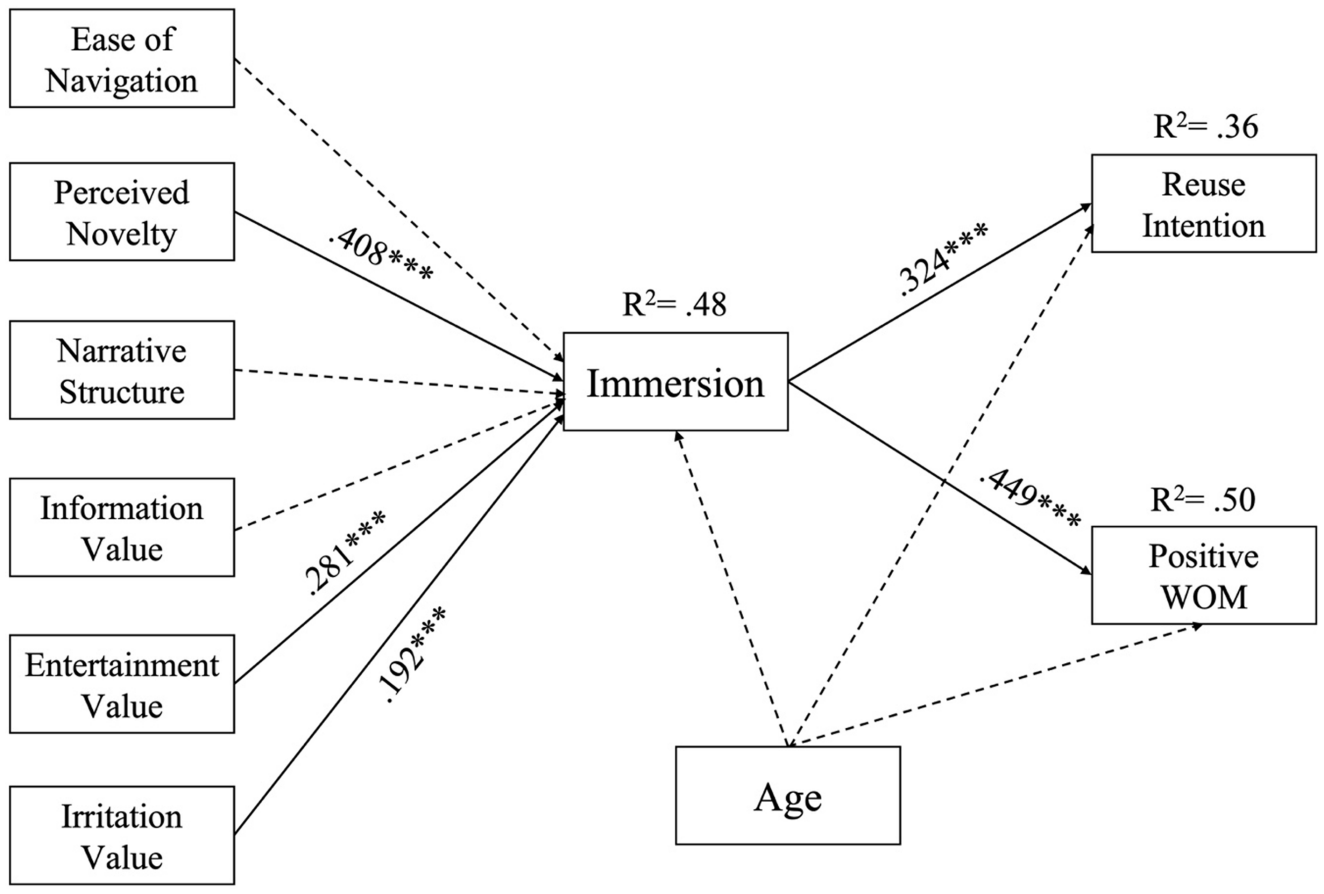
Structural model.

The results from the testing of the hypotheses and the answers to research questions are presented in Table 5 and Figure 1. The model explained 36% of the variance in reuse intention, 50% of the variance in positive word of mouth, and 48% of the variance in the immersive experience. Therefore, the model of the hypotheses was acceptable.

**Table 5:** Hypothesis testing results.

Hypotheses	Path coefficient	SE	*t*	*P*
H1a: IM → WOM	0.45	0.05	10.03	0.00
H1b: IM → RI	0.32	0.05	7.20	0.00
H2: EON→IM	0.02	0.06	0.32	0.75
H3: PN → IM	0.41	0.08	5.05	0.00
H4: NS → IM	0.09	0.08	1.20	0.23
RQa: InV → IM	−0.04	0.07	−0.66	0.51
RQb: EV → IM	0.28	0.08	3.52	0.00
RQc: IrV → IM	0.19	0.05	4.04	0.00

As expected, perceived immersive experience had a positive impact on positive word of mouth (*β* = 0.45, *SE* = 0.05, *t* = 10.03, *p* < 0.001) and on continuance intentions (*β* = 0.32, *SE* = 0.05, *t* = 7.20, *p* < 0.001) for short video applications, suggesting that H1a and H1b were supported by the data. Turning to the six components of the independent variables, immersion was positively related to perceived novelty (*β* = 0.41, *SE* = 0.08, *t* = 5.05, *p* < 0.001), entertainment value (*β* = 0.28, *SE* = 0.08, *t* = 3.52, *p* < 0.001), and irritation value (*β* = 0.19, *SE* = 0.05, *t* = 4.04, *p* < 0.001). However, the path coefficients of the other constructs, perceived ease of navigation (*β* = 0.02, not significant), narrative structure (*β* = 0.09, not significant), and information value (*β* = −0.04, not significant), did not affect immersion significantly. Therefore, H3 was supported, but H2 and H4 were not. The results indicate that RQb and RQc should be answered in the affirmative, while the answer to RQa should be negative.

We conducted a mediation analysis to determine whether the relationships between the independent variables and usage behavior had been mediated by immersion. The specific indirect associations were obtained through a bootstrap procedure that we ran 1,000 times, as suggested by Preacher and Hayes (2008). If the 95% confidence interval around the estimations of the indirect relationships did not include zero, the results confirmed a mediation relationship. The results (see Table 6) indicated that the mediation associations between perceived novelty, entertainment value, irritation value, and the two kinds of usage behavior through immersive experience were statistically significant.

**Table 6:** Mediation analysis results of immersive experience.

Mediation path	Coefficient	*t*	*P*	Bootstrap SE
EON→IM → WOM	0.01	0.32	0.75	0.01
EON→IM → RI	0.01	0.32	0.75	0.01
PN → IM → WOM	0.18	4.45	0.00	0.18
PN → IM → RI	0.13	4.09	0.00	0.13
NS → IM → WOM	0.04	1.19	0.23	0.04
NS → IM → RI	0.03	1.18	0.24	0.03
InV → IM → WOM	−0.02	−0.66	0.51	−0.02
InV → IM → RI	−0.01	−0.66	0.51	−0.01
EV → IM → WOM	0.13	3.30	0.00	0.13
EV → IM → RI	0.09	3.11	0.00	0.09
IrV → IM → WOM	0.09	3.78	0.00	0.09
IrV → IM → RI	0.06	3.56	0.00	0.06

## 5. Discussion

### 5.1. Theoretical significance

Research regarding immersive experiences on the internet and in human-computer interactions has lacked theoretical treatments of short video applications (Shrikant et al., 2020). This study focused on the design rather than the adverse effects of video consumption, such as media addiction and social isolation, in order to explain the practice of viewing videos for an extended time (Wegmann and Brand, 2016). We considered the large number of consumers who use mobile devices to access videos. This study has enriched immersion theory and extended the stimulus–organization–response model (Mehrabian and Russell, 1974) to the domain of technological affordances by developing a theoretical model for the formation and the influential mechanisms of the construct of immersive experience in the context of short videos. This model highlights the value of video content. Our results are novel in that we found that perceived novelty and content value (entertainment and irritation) affected immersion positively. Thus, the perceived situation affordance and the affective affordance of short videos, which subsequently influenced individuals’ reuse and recommendation intentions, were highlighted. These findings indicate that not all social media affordances are significant—it is necessary to analyze the impact of different affordances specifically and to modify the use of the concept of affordance that has emerged from the literature.

The main contributions of this study are as follows. First, earlier research has shown that short video applications have adapted algorithm technology to offer users personalized streams that reflect the characteristics of the user, the content, and the environment (Zhu and Cui, 2018). Our model accounted for clickthrough rates, reading times, likes, comments, and forwarding. In other words, short video applications quickly assess the viewing needs of users through AI, which explains why short videos may be viewed for longer than expected and why addictive behavior can occur (Shao, 2018). We found that perceived novelty and entertainment value were positively related to immersion. Thus, if the content of the recommended stream met the user’s standards of perceived novelty and entertainment value and if it enabled viewers to satisfy their innate needs and to confirm their medium-specific gratification expectations (Sundar and Limperos, 2013), it became easier to improve their immersive experiences.

However, a personalized recommendation mechanism can also induce irritation. The algorithm may push homogeneous and vulgar content at users, resulting in insufficient content diversity. In addition, researchers have postulated that short video platforms do not bind users strongly because their content orientation is incorrect. These platforms have also been reported to have numerous adverse effects on teenagers (Meng and Leung, 2021). Irritation value has been found to reduce immersion (Ducoffe, 1995, 1996). To our surprise, our results showed that the opposite was true. This inconsistency may be due to users’ dismissing content that they did not accept and expecting the next video to be different. However, the algorithm recorded such actions as instances of browsing and continued recommending similar content to the user.

Second, the platforms that we studied mostly offer user-generated content. Users are content producers, participants, and communicators; TikTok is a suitable example. Users can record 15 s of an original short music video and share their creative or humorous ideas. They can use video templates and props from the video library, and they only need to imitate and replicate exciting and straightforward actions to create interesting content. This functionality activates creativity and pushes users to explore the application further and to interact with it (Xu and Tayyab, 2021). The innovative and memorable content arouses a sense of novelty. Media users also expect to follow trends and to receive gratifying feedback (Vaterlaus and Winter, 2021). Short video caters to the entertainment needs of contemporary users, anytime and anywhere, bringing vivid sensations to users and creating positive and immersive experiences. Content of this kind, such as Facebook games with unusual components, has been associated with enhanced flow experiences (Liu and Shiue, 2014). In addition, our results suggest that there is not a significant relationship between informativeness and immersion. This conclusion is partially consistent with the notion that entertainment is a stronger motivator of engagement in online consumption than informativeness (Luo, 2002).

Third, immersive experiences affect usage behavior significantly. They encourage users to continue visiting a platform and to comment favorably on it, facilitating the rapid expansion of the short video user market. The success of short videos is not based solely on their format but also on the emotional involvement of users. This positive attitude is likely to translate into a positive evaluation of the platform. Users are also more willing to increase the frequency and duration of use as well as to intensify their future use intentions. These results agree with those from an earlier study (Ltifi, 2018). The author of that study examined the manner in which immersion influences the intention to use smartphones for e-commerce, corroborating the proposition that mobile applications are more attractive than desktop ones. Moreover, algorithms are thought to provide users with highly immersive experiences (Xu and Tayyab, 2021) and to encourage them to interact with equipment rather than with other individuals. Social interaction has been found to interrupt immersion and, by extension, to diminish its positive effect on satisfaction and loyalty (Sweetser and Wyeth, 2005). Our study has demonstrated that human–computer interactions can affect usage behavior.

### 5.2. Practical significance

This study can offer suggestions for the design of short video applications that can attract and keep the attention of consumers. To create more immersive experiences for users, designers should focus on novelty and entertainment value. Moreover, designers should create content that includes surprising, pleasurable, and original elements in order to generate more recommendations. At the same time, the features of short video applications should be updated constantly and in line with customer feedback so that browsing and content creation can become easier. As suggested in the literature, editing, and looping functionalities evoke emotions of vitality and positive feelings in users (Bernardi et al., 2018).

### 5.3. Limitations and future directions

This study has several limitations. First, it examined the typical characteristics of various short videos. Given the increasing variety of video applications, it is essential to subdivide short videos into types and content categories in order to identify the various social impacts of technological affordances. Second, our results indicated that irritating content affected immersion positively. Given that previous studies have arrived at the opposite finding, further research is warranted. Third, the data that we used was cross-sectional. Therefore, the results revealed only associations between the examined variables; an experimental design or the use of multiple measurement points over time would be necessary to infer causality confidently. Fourth, all data collected was self-reported by users. Self-reported measures of online media use remain crucial for communication research; however, previous studies have found low levels of measurement correspondence and tendencies of over-reporting for internet use (Scharkow, 2016; Araujo et al., 2017). To overcome possible biases, future investigators are advised to combine survey data with other sources, such as tracking data, event sampling, or qualitative data. Fifth, more than half of our participants were female. Although previous studies have shown that gender exerted no significant effects on immersion (Cuny et al., 2015) or social media use (Zhou and Zhang, 2019), we recognize that gender distribution may produce precise variability. Lastly, the data for this study was only collected from China. Considering the global development of short video applications and the differences among the dominant applications in different regions, we suggest that more researchers from different cultural backgrounds should explore the role of cultural factors.

## 6. Conclusion

We have developed a model to explore the influence of technological affordances on short video usage. A significant body of research has identified various technical aspects that may stimulate immersion. A novel finding from our study is that perception (including that of content and that of an application) and content value (entertainment and irritation) can influence immersion positively. Conversely, we found that ease of navigation, narrative structure, and the information value of short video applications did not influence immersion. Thus, the perceived situation affordance and affective affordance of short videos have been highlighted. Subsequently, they influence reuse and recommendation intentions. Thus, it is necessary to analyze the impact of different affordances and to modify the general definition of the concept of technological affordance that has emerged from the literature. These findings indicate that the appeal of a short video does not lie merely in the novelty of the format and its settings; what matters most is emotional engagement.

## Data availability statement

The raw data supporting the conclusions of this article will be made available by the authors, without undue reservation.

## Ethics statement

The studies involving human participants were reviewed and approved by the School of Journalism and Communication, Northwest University of Political Science and Law. Written informed consent to participate in this study was provided by the participants’ legal guardian/next of kin.

## Author contributions

YY and YH developed the study concept, gathered data, performed data analysis, and described the results. LL revised the manuscript and provided critical comments. YH supervised the research project. All authors contributed to the article and approved the submitted version.

## Funding

This work was supported by the Startup Fund for Young Faculty at SJTU (22×010500272).

## Conflict of interest

The authors declare that the research was conducted in the absence of any commercial or financial relationships that could be construed as a potential conflict of interest.

## Publisher’s note

All claims expressed in this article are solely those of the authors and do not necessarily represent those of their affiliated organizations, or those of the publisher, the editors and the reviewers. Any product that may be evaluated in this article, or claim that may be made by its manufacturer, is not guaranteed or endorsed by the publisher.
